# Prevalence of Mental Disorders in the South-East of Spain, One of the European Regions Most Affected by the Economic Crisis: The Cross-Sectional PEGASUS-Murcia Project

**DOI:** 10.1371/journal.pone.0137293

**Published:** 2015-09-22

**Authors:** Fernando Navarro-Mateu, Mª José Tormo, Diego Salmerón, Gemma Vilagut, Carmen Navarro, Guadalupe Ruíz-Merino, Teresa Escámez, Javier Júdez, Salvador Martínez, Ron C. Kessler, Jordi Alonso

**Affiliations:** 1 Unidad de Docencia, Investigación y Formación en Salud Mental (UDIF-SM), Servicio Murciano de Salud. Departamento de Psicología Básica y Metodología, Universidad de Murcia, Murcia, Spain; 2 IMIB-Arrixaca, Murcia, Spain; 3 CIBER de Epidemiología y Salud Pública (CIBERESP), Murcia, Spain; 4 Servicio de Epidemiología, Consejería de Sanidad y Política Social, Murcia, Spain; 5 Departamento de Ciencias Sociosanitarias, Universidad de Murcia, Murcia, Spain; 6 IMIM-Institut Hospital del Mar d´Investigacions Médiques, Barcelona, Spain; 7 Fundación para la Formación e Investigación Sanitarias (FFIS) de la Región de Murcia, Murcia, Spain; 8 IMIB BIOBANC-MUR, Biobanco-HUVA-AECC-FFIS, Murcia, Spain; 9 Sociedad Española de Patología Digestiva, Fundación Española del Aparato Digestivo, Madrid, Spain; 10 Department of Health Care Policy, Harvard Medical School, Boston, Massachusetts, United States of America; 11 Departamento de Salud y Ciencias Experimentales, Universidad Pompeu Fabra, Barcelona, Spain; Peking University, CHINA

## Abstract

**Background:**

To describe the lifetime and 12-month prevalence, severity and age of onset distribution of DSM-IV (Diagnostic and Statistical Manual of Mental Disorders) disorders and to explore the association between socio-demographic variables and economic stressors with mental disorders during the economic crisis in the general population of Murcia (Spain).

**Methods and Findings:**

The PEGASUS-Murcia Project is a cross-sectional face-to-face interview survey of a representative sample of non-institutionalized adults in Murcia administered between June 2010 and May 2012. DSM-IV disorders were assessed by the Composite International Diagnostic Interview (CIDI 3.0). Main outcome measures were lifetime and 12-month prevalence of Anxiety, Mood, Impulse and Substance Disorders, Severity and Age of Onset. Sociodemographic variables and stressful economic life events during the preceding 12 months were entered as independent variables in a logistic regression analysis. A total of 2,621 participants (67.4% response rate) were interviewed, 54.5% female, mean age 48.6 years. Twelve-month prevalence (95%CI) of disorders: anxiety 9.7% (7.6–12.2), mood 6.6% (5.5–8.1), impulse 0.3% (0.1–1.2) and substance use 1.0% (0.4–2.4) disorders. Lifetime prevalence: anxiety 15.0% (12.3–18.1), mood 15.6% (13.5–18.1), impulse 2.4% (1.4–4.0) and substance use 8.3% (6.2–11.0) disorders. Severity among 12-month cases: serious 29.2% (20.8–39.4), moderate 35.6% (24.0–49.1) and mild severity 35.2% (29.5–41.5). Women were 3.7 and 2.5 times more likely than men to suffer 12-month anxiety and mood disorders, respectively. Substance use was more frequent among men. Younger age and lower income were associated with higher prevalence. Respondents exposed to multiple and recent economic stressors had the highest risk of anxiety disorders.

**Conclusions:**

Mental disorders in the adult population of Murcia during the economic crisis were more prevalent and serious than those in previous estimates for Spain. Prevalence was strongly associated with exposure to stressors related to the economic crisis.

## Background

The World Mental Health (WMH) Survey Initiative is a World Health Organization (WHO) initiative designed to carry out epidemiological surveys on mental disorders in a number of countries from all major regions of the world and to overcome the main problems associated with previous community studies (cross-national comparability, selection procedures of the target population and different diagnostic instruments) [1–3]. The enormous body of comparative cross-national data on the epidemiology of mental disorders all over the world suggests the existence of wide variation in the 12-month prevalence of DSM-IV (Diagnostic and Statistical Manual of Mental Disorders) disorders, from a high of 26.4% in the United States to a low of 4.3% in Shangai [4]. The lifetime prevalence of having at least one mental disorder also varies across the WMH surveys, from a high of 47.4% in the United States to a low of 12.0% in Nigeria [5]. As part of this epidemiological effort, the European Study of the Epidemiology of Mental Disorders (ESEMeD) project was designed to collect data from representative samples of the adult population in six European countries: Belgium, France, Germany, Italy, the Netherlands and Spain [6]. In Europe, twelve-month and lifetime prevalence of any disorder were 10% and 25% [7] and, in Spain, 9.2% [7] and 19.4% [5], respectively.

Since 2007, most European countries have experienced one of the longest economic crises in recent memory and Spain is one of the most seriously affected nations. The Spanish unemployment rate has increased dramatically from an annual average of 8% in 2007 to 24.5% in 2014, the second highest in Western Europe [8]. Evidences suggest that this crisis has led to a substantial increase in the prevalence of psychological disorders among men [9] as well as to an increase in suicidal behavior [10,11] and a substantial increase in the frequency of treatment of mental disorders among primary care attendees [12]. However, existing studies of these effects are limited in several ways [13,14]. Many have used proxies of mental disorders, such as suicide [10,11,15] whilst others have used administrative databases [9,10,16], thus increasing the risk of ecological fallacy. Also, most studies assessed non-representative samples of the general population [10,12,16]. At an international level, it has been suggested that there is still a lack of population-level research on the relationship between the economic consequences of a recession and specific mental disorders [14,17,18]. In this heterogeneous scenario, new epidemiological surveys are justified specifically in areas where mental health care planning, management and decision-making are made [19]. In Spain, several differences between the Autonomous Communities in important aspects related to mental health have recently been described [20–23]. Moreover, the field work of the latest epidemiologic study data of a representative sample of the general population for the evaluation of common mental disorders in Spain took place between 2001 and 2002 in the context of the ESEMeD project, five years before the beginning of the economic crisis [6,24].

The PEGASUS-Murcia (“Psychiatric Enquiry to General Population in Southeast Spain-Murcia”) project was a WMH survey carried out between 2010 and 2012 designed to obtain regional data of the prevalence, burden and treatment of mental disorders in a representative sample of the general adult population of Murcia. It was carried out in order to provide a need assessment for planning new regional mental health policies as well as to compare results with national data from Spain, Europe and all other countries participating in the WMH Survey Initiative [25]. The aims of this study were to identify the lifetime and 12-month prevalence, the level of severity and age of onset of DSM-IV disorders and to explore the association of several socio-demographic variables and the exposure to economic stress, with mental disorders during the economic crisis in Murcia (Spain), using data from the PEGASUS-Murcia project.

## Methods

The PEGASUS-Murcia project is a cross-sectional survey based on a representative sample of the adult and non-institutionalized general population of the Murcia Region. The protocol with further description of the sampling frame, selection and weighting procedures is described in more detail elsewhere [25]. Murcia is one of the 17 Autonomous Communities of Spain and is located in the southeast of the country on the Mediterranean coast, with a population of 1,424,063 inhabitants at the time of the survey [26], almost a third of them (30.7%) living in the capital. The target population was defined as persons aged 18 or older residing in Murcia, not living in institutions and registered in PERSAN, a periodically up-dated regional registry containing all residents as there was a universal health coverage by the time of the survey. Murcia is divided into nine Health Care Areas and a stratified, multistage, clustered health area, probability random sample design was used. Exclusion criteria were: i) Confirmed irretrievable contact errors (e.g. telephone number and address); ii) Institutionalized individuals (e.g. in prison, in a hospital or in another institution) or those living outside the Autonomous Community during the survey field work; and iii) individuals not able to understand the Spanish language or not able to conduct the questionnaire due to his/her physical or mental condition.

### Assessment of lifetime and 12-month diagnosis and persistence: The survey questionnaire

The questionnaire used in the PEGASUS-Murcia project is a revised version of the WHO Composite International Diagnostic Interview (CIDI 3.0, hereafter referred to as CIDI), specifically adapted for use in Spain [27]. The CIDI is a comprehensive, highly-structured interview specifically designed by the World Health Organization (WHO) for the purpose of ascertaining diagnoses of mental illnesses for comparative research of the epidemiology of mental illnesses throughout the world [28]. It also includes specific information on the severity of the disorders, symptoms, disability, quality of life, use of services and medication and several risk factors. In order to optimize the duration of the interview, the WMH questionnaire was divided into two parts with questions in Part 1 administered to all respondents and those in Part 2 administered only to a subsample of individuals who followed the long path of the interview (all respondents with Part 1 lifetime core disorders and a probability sub-sample of other Part 1 respondents, with a weighting used to adjust for the under-sampling of respondents without disorders to maintain the representativeness of the sample to the population). Questionnaire pathways are described elsewhere [25]. Face-to-face interviews were carried out between June 2010 and May 2012 by certified lay interviewers using CAPI (Computer Assisted Personal Interviewing).

Prevalence estimates of mental disorders were determined on the basis of whether respondents’ past or current symptomatology met the 12-month and/or lifetime diagnostic criteria for a DSM-IV disorder. The ratio of the 12-month prevalence to lifetime prevalence is considered as an indirect indicator of persistence or progression of the illness with higher ratios suggesting higher persistence throughout the life course [29]. In this paper, 12-month and lifetime prevalence rates are presented, as well as correlates of 12-month mental disorders according to DSM-IV (i.e. Mood Disorders-including major depression, bipolar and dysthymia-, Anxiety Disorders-generalized anxiety disorder, social phobia, specific phobia, post-traumatic stress disorder, agoraphobia without panic, panic disorder, obsessive compulsive disorder and adult separation anxiety disorder-, Substance Disorders–alcohol and drug abuse and/or dependence- and Impulsive Disorder–oppositional-defiant, conduct and attention deficit disorders). Other disorders included in the CIDI questionnaire, such as eating disorders and childhood disorders were not evaluated in this issue because they were only assessed in a small subsample of respondents. All diagnoses were considered with organic exclusions and without diagnostic hierarchy rules with the exception of major depressive disorder, dysthymia, general anxiety disorder and oppositional-defiant disorder. For substance use disorders, abuse was defined with or without dependence in recognition of abuse being a stage in the progression to dependence.

### Severity of 12-month disorders

Respondents were categorized as having severe mental illnesses [30] if they were diagnosed with 12-month bipolar I, attempted suicide in the last 12-months and had any 12-month diagnosis, had substance dependence with physiological symptoms or had more than one 12-month diagnosis and a high level of impairment on any of the Sheehan scales [31], including disability in work role performance, household maintenance, social life, and intimate relationships. Among those who were not categorized as severe, respondents were labeled moderate if they had at least one disorder and a moderate level of impairment or they had substance dependence without physiological signs. The remaining respondents with any 12-month disorder were categorized as mild.

### Age of onset and socio-demographic variables

Retrospective age-of-onset reports were obtained in the WMH-CIDI using a series of questions designed to avoid the implausible response patterns obtained when using the standard CIDI age-of-onset question [32]. Socio-demographic variables included: age at interview (categorized as <35, 35–49, 50–64, 65+); sex; completed years of education (four categories: None or primary: 0–7 years; Basic: 8–11 years; Secondary: 12–15 years and College: 16 or more years of education); marital status (married-cohabitating, separated-widowed-divorced, never married); and family income. The latter is the sum of all pre-tax income in the past 12 months, including salaries earned by all members of the household plus all sources of other income (e.g., government transfers, pensions and investment income). Respondents report each income component in a range of euros (e.g., 14,000–14,999 €), which we converted to the midpoint of the range (e.g., 14,500 € in the above example) to simplify calculations. If a respondent did not report any single component of income, regression based imputation was used. Per capita income was then calculated for each family. This is the family income divided by the number of people in the household, as reported in the household listing. In order to compare income across countries within the WMH Surveys, we created a four-category income scale. A respondent was assigned a category on this scale based on the per capita income of the respondent's family divided by the median income for Spain. The household is in the low, low-average, high-average or high categories if this ratio is 0.5 or less, >0.5 to 1.0, >1.0 to 2.0, or over 2.0, respectively. Employment status was categorized in 6 categories (working, student, homemaker, retired/disabled, unemployed and others).

### Stressful life events during the preceding 12 months

Stressful life events related to the economic crisis during the preceding 12 months were measured using the LTE-Q (List of Threatening Experiences-Questionnaire version), a brief inventory of 12 life events categories with considerable long-term contextual threat[33]. This scale was adapted in two ways. Firstly, the retrospective exposure to each of the events for the period of the preceding 12 months was asked (“Now we are going to ask you if you suffered any of the following events in the last 12 months …”), whether it had occurred (score = 1) or not (score = 0) was recorded. Secondly, for the events occurred, additional questions were asked to assess the emotional impact (0, no event; 1, event that left the individual fairly calm; 2, event that left the individual shocked but able to cope; 3, event that left the individual rather overwhelmed) and degree of life change (0, no event; 1, event occurred but has not changed life in any way; 2, event occurred and has changed life; 3, changed a great deal) [34]. For the present project, only the 3 events from the LTE-Q related to any economic problems were included in the analyses: “Have you been unemployed or looking for work for over a month without success?”; “Have you been made redundant?”; and “Have you had a severe financial crises or major economic problems?”. Three continuous aggregated economic life-event scores were then constructed. The first one, known as the Economic Crisis Score (ECS), was calculated by adding the score for each event occurred (range: 0–3). The second and the third ones, named Emotional Impact Score (EIS) and Life-Change Score (LCS) respectively, by classifying the answers of the emotional impact and life change of the event occurred into different categories according to the level of impact independently of the number of events (0, no event; 1, any of the three events occurred but neither affected or changed the life of the interviewee; 2, any of the three events occurred and at least one of them shocked or changed the individual; 3, any of the three events occurred overwhelmed or drastically changed the life of the individual). Finally, because ECS showed near-perfect correlations with the other two scores, EIS (Spearman`s rho r = 0.99) and LCS (r = 0.99) and so did EIS and LCS (r = 0.99), only ECS was considered in subsequent analyses.

### Analysis methods

Weights were used to adjust for differential probabilities of selection for each Health Care Area, Health Center and demographic strata. An additional part 2 weight to adjust for oversampling of high risk individuals was used to restore distribution of the general population in terms of sex, age and area. Weighting procedures are described in more details elsewhere [25]. Prevalence estimates are provided and expressed in absolute numbers and weighted percentages with 95% confidence intervals (CI). Lifetime prevalence was estimated as the weighted percentage of respondents fulfilling DSM-IV criteria for a given disorder up to their age at interview. Persistence was calculated indirectly as the ratio between 12-month and lifetime prevalence. All the statistical tests were carried out using the “*svy*” command. Correlates of mental disorders were investigated by multiple logistic regression analyses, with any disorder, mood and anxiety disorder as the dependent variable. The small number of individuals with impulse or substance disorder prevented them from the analyses. Socio-demographic characteristics (sex, age, marital status, education, employment status) and ECS were entered as independent variables. Projected life-time risk was estimated using the two-part actuarial method implemented in SAS version 8.2 using the age of onset information. All analyses were weighted and standard errors, confidence intervals and inference tests were obtained using the Taylor series linearization method [35] implemented in the STATA software Version 10.0, using the “*svy*” command with weights specified as “probability weights” to adjust for the effects of weighting and clustering on the precision of estimates [36]. Written informed consent was obtained from all participants. The protocol was approved by the Clinical Research Ethics Committee of the University Hospital *Virgen de la Arrixaca* of Murcia and the database of personal information was registered with the National Data Protection Agency. The present study has been written in accordance with the STROBE (Strengthening The Reporting of Observational Studies in Epidemiology) statement guidelines (S1 Table: Specific STROBE Checklist) [37].

## Results

**Fig 1:**
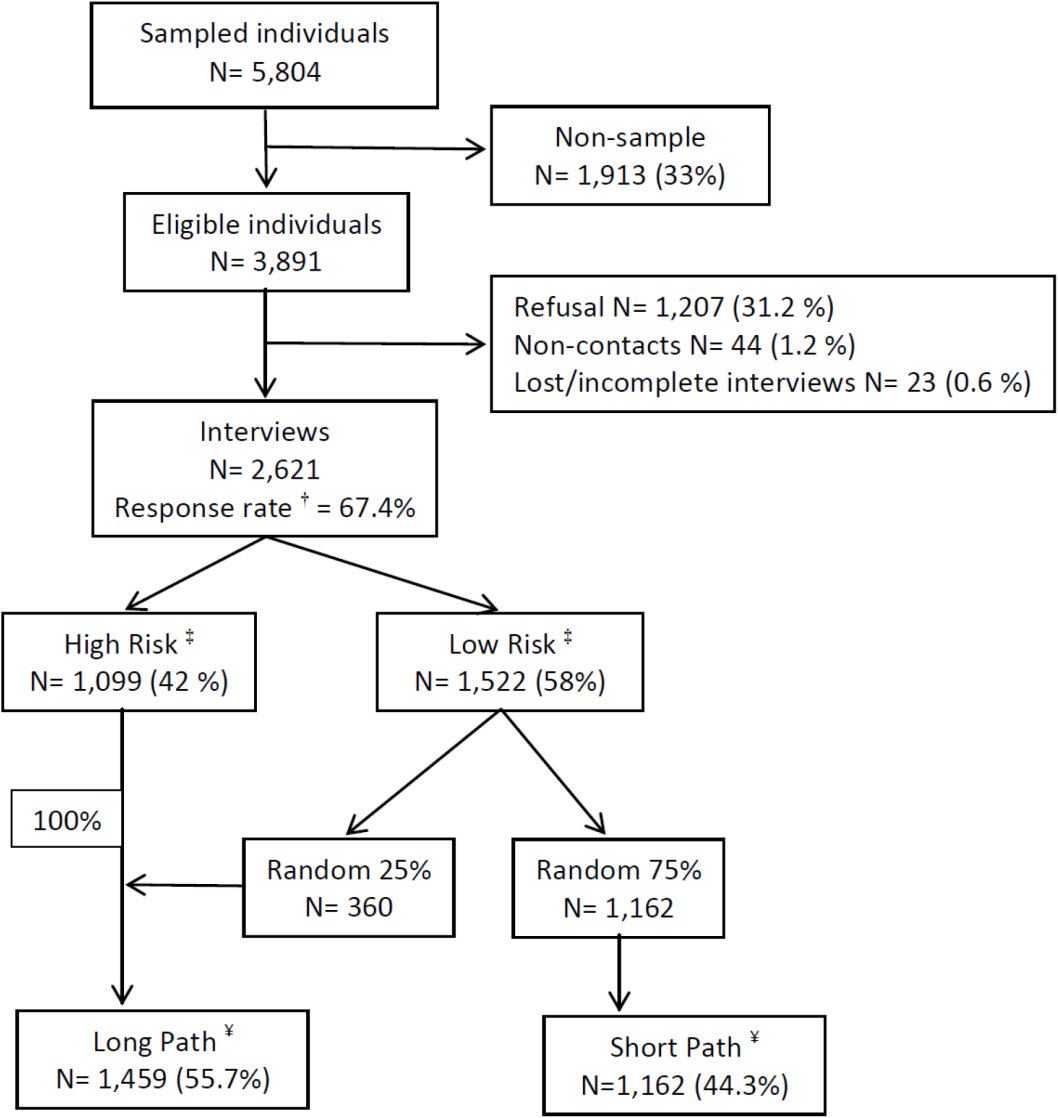
Description of the final sample in the PEGASUS-Murcia project. ^†^ The response rate is defined as: ***(completed interviews) / (total released respondent sample cases–respondent nonsample cases)*.** ^‡^ **High risk individuals**: those who have positively answered a number of specific questions related to mood and anxiety disorders in the screening section. **Low risk individuals**: those without symptoms related to mood and anxiety disorders in the screening section. ^¥^ **Long Path inclusion criteria**: a) all high risk individuals and b) a random subsample of 25% of the low risk individuals. The remaining 75% of respondents without screening symptoms not randomly selected for the long path followed the **Short Path** of the questionnaire

A total number of 2,621 participants, with an overall response rate of 67.4% (range according to Health area from 62% to 70%), were interviewed (Fig 1). Description of the socio-demographic characteristics of the participants is presented in Tables 1 and 2. Married or cohabitating was the most frequent marital status; slightly more than 50% were in the middle levels of education and with the average level of income. Almost three quarters of the population lived in an urban area geographical area (with a population of 10000 inhabitants or more). Almost half of the population was working and only 10.7% are unemployed at the time of the interview. Over the last 12 months, almost 6% had been redundant from their job, 15% had been unemployed or looking for work for a month without success and 10% had had a severe financial crises or major economic problems. Almost a fifth of the sample had suffered an economic stressor over the last 12 months, with 63% of them with a single stressor and 7% with three of them. The comparison with available census data (sex, age and Health Care Areas) suggests that our sample is representative of the general population of the Region of Murcia.

**Table 1:** Sociodemographic distribution of the PEGASUS-Murcia sample compared to general population^$^.

	Interviewees		Census^#^	
	N	Weighted %	N	%
**Sex** ^*^				
Male	1 192	50.5	581493	50.3
Female	1 429	49.5	574798	49.7
**Age** ^*^ **(mean)**		(48.59)		(47.0)
18–24	179	8.0	125122	10.8
25–34	431	20.3	254938	22.0
35–49	841	31.6	353056	30.5
50–64	635	21.1	221946	19.2
65+	535	19.0	201229	17.4
**Health Care Areas** ^*^				
I	456	18.0	201497	17.4
II	397	20.0	227413	19.7
III	412	12.0	136584	11.8
IV	115	5.0	59975	5.2
V	92	4.1	47664	4.1
VI	517	17.4	202528	17.5
VII	404	13.3	158857	13.7
VIII	159	6.7	78174	6.8
IX	69	3.6	43599	3.8
**Marital status** ^*^				
Married/Cohabitating	1 879	71.1	-	-
Separated/Widowed/Divorced	318	10.9	-	-
Not Married	424	18	-	-
**Education** ^*^ ^**#**^				
None or Primary	671	24.1	-	-
Basic	830	31.8	-	-
Secondary	599	24.4	-	-
College	521	19.6	-	-
**Geographical area** ^*^				
Mid-size urban (2000–10000)	606	21.3	-	-
Rural (<2000)	129	2.5	-	-
Urban (> = 10000)	1 886	76.2		

^$^ Only sociodemographic variables used in post-stratification of weight are presented.

^*^ Weighted Percentages calculated using part 1 weights

^**^ Weighted Percentages calculated using part 2 weights

^**#**^ Centro Regional de Estadística de Murcia; Padrón 2010

# Completed years of education (four categories: None or primary: 0–7 years; Basic: 8–11 years; Secondary: 12–15 years and College: 16 or more years of education).

**Table 2:** Sociodemographic distribution (continuation) of the PEGASUS-Murcia sample compared to general population^$^.

	Interviewees	
	N	Weighted%
**Income** ^**^		
Low	370	25.0
Low-Average	452	30.1
High-Average	384	27.2
High	253	17.8
**Employment** ^*^		
Working	1 331	52.0
Student	106	4.6
Homemaker	329	11.7
Retired/disabled	549	19.2
Unemployed	252	10.7
Other	54	1.9
**Suffered any of the following events during the last 12 months. . .**		
**Unemployed or looking for work for over a month without success**		
No	2 238	85.0
Yes	378	15.0
**Fired from work?**		
No	2 482	94.1
Yes	133	5.9
**Severe financial crises or major economic problems**		
No	2 373	90.3
Yes	241	9.7
**12-month ECS** ^**&**^		
0	2 084	78.9
1	342	13.3
2	152	6.2
3	35	1.6

^$^ Only sociodemographic variables used in post-stratification of weight are presented.

^*^ Weighted Percentages calculated using part 1 weights

^**^ Weighted Percentages calculated using part 2 weights

^**#**^ Centro Regional de Estadística de Murcia; Padrón 2010

# Completed years of education (four categories: None or primary: 0–7 years; Basic: 8–11 years; Secondary: 12–15 years and College: 16 or more years of education).

† Family income is defined as a four-category income scale calculated as the ratio of family income in the past 12 months divided by the median income for Spain. Low income is defined as less than or equal to 0.5, low average as 0.5 to 1.0, high average as 1.0 to 2.0, and high as over 2.0.

^&^ 12-month ECS (12-month Economic Crisis Score) as the sum of the score of the three events suffered during the last 12 months. Range value from 0 to 3.

### Lifetime and 12-month prevalence and persistence

Table 3 shows that a third of the population fulfilled the criteria of a lifetime presence of any mental disorder, most of them with only one disorder, with slightly more than 16% having experiencing it in the past 12 months. Nearly 15% of the population reported a lifetime history of anxiety disorders, a similar percentage mood disorders, 2.4% an impulse disorder and almost 8% a substance abuse disorder. Within the period of 12 months preceding the interview, 9.7% met the criteria for anxiety disorders, 6.6% for mood disorders, 0.3% for an impulse disorder and 1% for a substance abuse disorder. Major depression, alcohol abuse with or without dependence and specific phobia were the three most common psychiatric disorders.

**Table 3:** Twelve-month and lifetime prevalence and persistence of mental disorders in the PEGASUS-Murcia project (weighted proportions and 95%CI).

				Lifetime	Prevalence			12 Month	Prevalence	Persistence	(12 month /	Lifetime)
Disorder			Total	Male	Female		Total	Male	Female	Total	Male	Female
Group	Disorder	N	% 95% CI	% 95% CI	% 95% CI	N	% 95% CI	% 95% CI	% 95% CI	% 95% CI	% 95% CI	% 95% CI
**Anxiety**	Panic Disorder*	41	1.6(0.9;2.7)	1.3(0.5;3.5)	1.9(1.3;2.6)	19	0.7(0.5;1.1)	0.3(0.1;1.0)	1.2(0.9;1.6) †	45.7(31.6;60.6)	20.3(3.8;62.2)	63.7(49.3;76.0)
**Disorder**	Generalized Anxiety Disorder*	130	4.7(3.1;7.0)	1.8(1.1;3.1)	7.6(5.2;11.1) ‡	77	3.0(1.8;5.0)	1.3(0.6;2.9)	4.7(3.1;7.3) ‡	64.4(53.1;74.3)	73.2(32.6;93.9)	62.3(53.9;70.0)
	Social Phobia*	43	1.7(1.2;2.4)	0.8(0.3;1.9)	2.6(1.6;4.2) †	33	1.2(0.8;1.8)	0.6(0.2;1.5)	1.8(0.9;3.3)	67.7(40.1;86.8)	70.9(27.4;94.1)	66.8(31.6;89.7)
	Specific Phobia*	137	5.4(4.4;6.5)	2.6(1.5;4.5)	8.2(7.3;9.2) ‡	118	4.7(3.8;5.7)	2.2(1.2;4.3)	7.1(6.0;8.5) ‡	86.9(80.1;91.7)	86.4(67.2;95.2)	87.1(73.4;94.3)
	Agoraphobia without Panic*	12	0.5(0.3;0.8)	0.0(na;na)	1.0(0.6;1.6)	7	0.2(1.0;0.6)	0.0(na;na)	0.5(0.2;1.2)	49.8(13.0;86.8)	0.0(na;na)	49.8(13.0;86.8)
	Post-Traumatic Stress Disorder**	65	2.8(1.9;4.1)	1.9(1.3;2.8)	3.7(2.3;5.9) †	26	0.9(0.5;1.7)	0.6(0.2;1.9)	1.2(0.7;2.1)	32.1(16.4;53.4)	31.9(8.9;69.3)	32.3(18.5;50.0)
	Adult separation anxiety disorder*	13	0.5(0.3;1.0)	0.4(0.2;0.9)	0.6(0.2;2.1)	0	0.0(na;na)	0.0(na;na)	0.0(na;na)	0.0(na;na)	0.0(na;na)	0.0(na;na)
	Obsessive Compulsive Disord.***	5	0.4(0.1;1.7)	0.0(na;na)	0.9(0.2;3.6)	3	0.3(0.1;1.4)	0.0(na;na)	0.6(0.1;2.9)	70.7(19.6;96.0)	0.0(na;na)	70.7(19.6;96.0)
	Any Anxiety Disorder**	342	15.0(12.3;18.1)	7.4(5.0;10.8)	22.6(18.6;27.3) ‡	227	9.7(7.6;12.2)	4.1(2.6;6.6)	15.3(12.0;19.3) ‡	64.6(57.3;71.2)	55.6(35.0;74.5)	67.5(61.8;72.8)
**Mood**	Dysthymia*	15	0.6(0.3;1.5)	0.5(0.1;1.8)	0.8(0.3;2.4)	9	0.5(0.2;1.4)	0.4(0.1;1.8)	0.6(0.2;2.3)	77.2(39.2;94.7)	74.3(21.3;96.9)	78.9(35.1;96.3)
**Disorder**	Major Depressive Disorder*	370	13.8(12.1;15.6)	9.4(7.8;11.3)	18.2(15.9;20.7) ‡	154	6.0(5.1;7.0)	3.3(2.3;4.7)	8.7(7.0;10.7) ‡	43.4(39.0;48.0)	35.4(23.2;49.8)	47.7(41.4;54.1)
	Bipolar Disorder (Broad)*	42	1.8(1.0;3.3)	1.9(1.0;3.7)	1.7(0.7;3.9)	19	0.7(0.3;1.4)	0.5(0.2;1.2)	0.9(0.3;2.4)	37.6(21.4;57.1)	24.1(7.7;55.0)	53.1(36.0;69.5)
	Any Mood Disorder*	415	15.6(13.5;18.1)	11.4(9.3;13.9)	19.9(17.3;22.8) ‡	173	6.6(5.5;8.1)	3.8(2.6;5.4)	9.6(7.6;11.9) ‡	42.5(37.8;47.4)	33.1(23.4;44.6)	48.1(42.0;54.1)
**Impulse**	Oppositional-Defiant Disorder**	13	0.6(0.3;1.6)	0.8(0.3;2.4)	0.5(0.1;2.0)	1	0.0(0.0;0.1)	0.0(na;na)	0.0(0.0;0.2)	1.6(0.1;20.9)	0.0(na;na)	4.1(0.2;52.5)
**Disorder**	Conduct Disorder**	10	0.7(0.3;2.0)	1.0(0.4;2.7)	0.5(0.1;3.1)	1	0.0(0.0;0.1)	0.0(na;na)	0.0(0.0;0.2)	1.4(0.1;21.1)	0.0(na;na)	4.2(0.1;62.4)
	Attention Deficit Disorder**	19	1.5(0.7;3.1)	1.8(0.5;6.1)	1.2(0.7;1.9)	5	0.3(0.1;1.2)	0.3(0.1;1.3)	0.4(0.1;1.7)	23.0(3.8;68.9)	17.4(1.9;69.4)	31.7(2.9;87.7)
	Any Impulse-Control Disorder**	35	2.4(1.4;4.0)	2.7(1.1;6.4)	2.1(1.5;3.0)	6	0.3(0.1;1.2)	0.3(0.1;1.3)	0.4(0.1;1.7)	14.4(3.7;42.1)	11.4(2.1;43.7)	18.3(3.4;58.5)
**Substance**	Alcohol Abuse with/without Depend.**	92	6.3(5.0;7.9)	11.3(8.7;14.7)	1.2(0.6;2.4) ‡	14	0.9(0.3;2.3)	1.7(0.6;4.4)	0.1(0.0;0.2) ‡	14.0(5.3;32.3)	15.0(5.4;35.4)	4.4(1.0;17.5)
**Disorder**	Alcohol Abuse without Depend.**	73	4.9(3.6;6.6)	8.6(6.3;11.8)	1.1(0.6;2.1) ‡	12	0.6(0.2;2.0)	1.2(0.4;3.8)	0.1(0.0;0.2) ‡	13.2(3.6;38.0)	14.3(3.7;41.6)	4.7(1.0;18.8)
	Alcohol Dependence**	20	1.5(0.7;3.1)	2.8(1.2;6.3)	0.1(0.0;1.0) ‡	3	0.3(0.1;1.3)	0.6(0.1;2.5)	0.0(na;na)	20.6(3.9;62.6)	21.2(3.9;63.9)	0.0(na;na)
	Drug Abuse with/without Depend.**	46	3.7(2.3;5.8)	6.0(3.3;10.7)	1.3(0.4;4.8) †	3	0.1(0.0;0.6)	0.2(0.0;0.9)	0.0(0.0;0.3) ‡	3.1(0.5;16.9)	3.2(0.6;16.0)	2.9(0.2;29.3)
	Drug Abuse without Depencence**	29	2.4(1.3;4.5)	3.7(1.7;8.1)	1.1(0.3;4.1)	1	0.0(0.0;0.1)	0.0(na;na)	0.0(0.0;0.3)	0.8(0.1;6.5)	0.0(na;na)	3.5(0.2;35.2)
	Drug Dependence**	17	1.2(0.6;2.5)	2.3(1.0;4.8)	0.2(0.1;0.7) ‡	2	0.1(0.0;0.5)	0.2(0.0;0.9)	0.0(na;na)	7.6(0.8;44.7)	8.4(0.9;47.4)	0.0(na;na)
	Any Substance Use Disorder**	115	8.3(6.2;11.0)	14.3(10.3;19.5)	2.2(1.0;5.0) ‡	17	1.0(0.4;2.4)	1.9(0.8;4.5)	0.1(0.0;0.4) ‡	11.9(4.4;28.0)	13.0(4.7;31.2)	4.1(0.5;27.1)
**Any**	Any**	678	33.2(29.2;37.4)	27.4(21.5;34.2)	39.0(35.5;42.5) ‡	364	16.3(13.7;19.2)	9.3(6.6;13.1)	23.4(20.4;26.6) ‡	49.2(42.3;56.1)	34.0(23.1;46.9)	60.0(55.9;63.9) ‡
**Disorder**	0 Disorders**	781	66.9(62.6;70.8)	72.6(65.8;78.5)	61.0(57.5;64.5) ‡	1095	83.7(80.8;86.3)	90.7(86.9;93.5)	76.6(73.4;79.6) ‡	na(na;na)	na(na;na)	na(na;na)
	1 Disorder**	433	21.0(18.9;23.3)	15.2(12.6;18.2)	26.9(24.0;30.1) ‡	273	12.2(10.8;13.6)	6.8(5.2;9.0)	17.5(14.6;20.9) ‡	42.4(37.3;47.7)	25.4(17.7;35.1)	52.1(46.2;58.0) ‡
	2 Disorders**	153	7.9(6.3;9.7)	8.2(6.1;11.0)	7.5(4.9;11.3)	68	3.2(1.8;5.6)	2.3(0.9;5.4)	4.2(2.4;7.2)	24.9(13.2;41.9)	15.4(5.8;34.9)	35.5(20.7;53.6) †
	3+ Disorders**	92	4.3(2.6;7.0)	4.0(2.0;8.1)	4.6(3.1;6.8)	23	0.9(0.6;1.5)	0.2(0.1;0.8)	1.6(1.1;2.6) ‡	21.8(12.0;36.2)	5.9(1.2;24.0)	36.0(19.7;56.3) ‡

* Part1 sample, prevalence calculated using part 1 weights.

** Part2 sample, prevalence calculated using part 2 weights.

*** Part3 sample, 33% random draw of PART 2 subsample using part 2 weights.

† p-value for sex-differences ≤ 0.05

‡ p-value for sex-differences ≤ 0.001.

Persistence ratios varied widely from 11.9% for any substance use disorder to 64.6% for any anxiety disorder, with 49.2% as the total persistence for any disorder. Individually, specific phobia (86.9%), dysthymia (77.2%), obsessive compulsive disorder (70.7%), social phobia (67.7%) and GAD (64.4%) had a ratio of over one half. There were no sex differences in any specific disorder or group of them, except for the number of disorders presented with a higher percentage of persistence among women, compared to men (Table 3).

### Age of onset distributions

**Fig 2:**
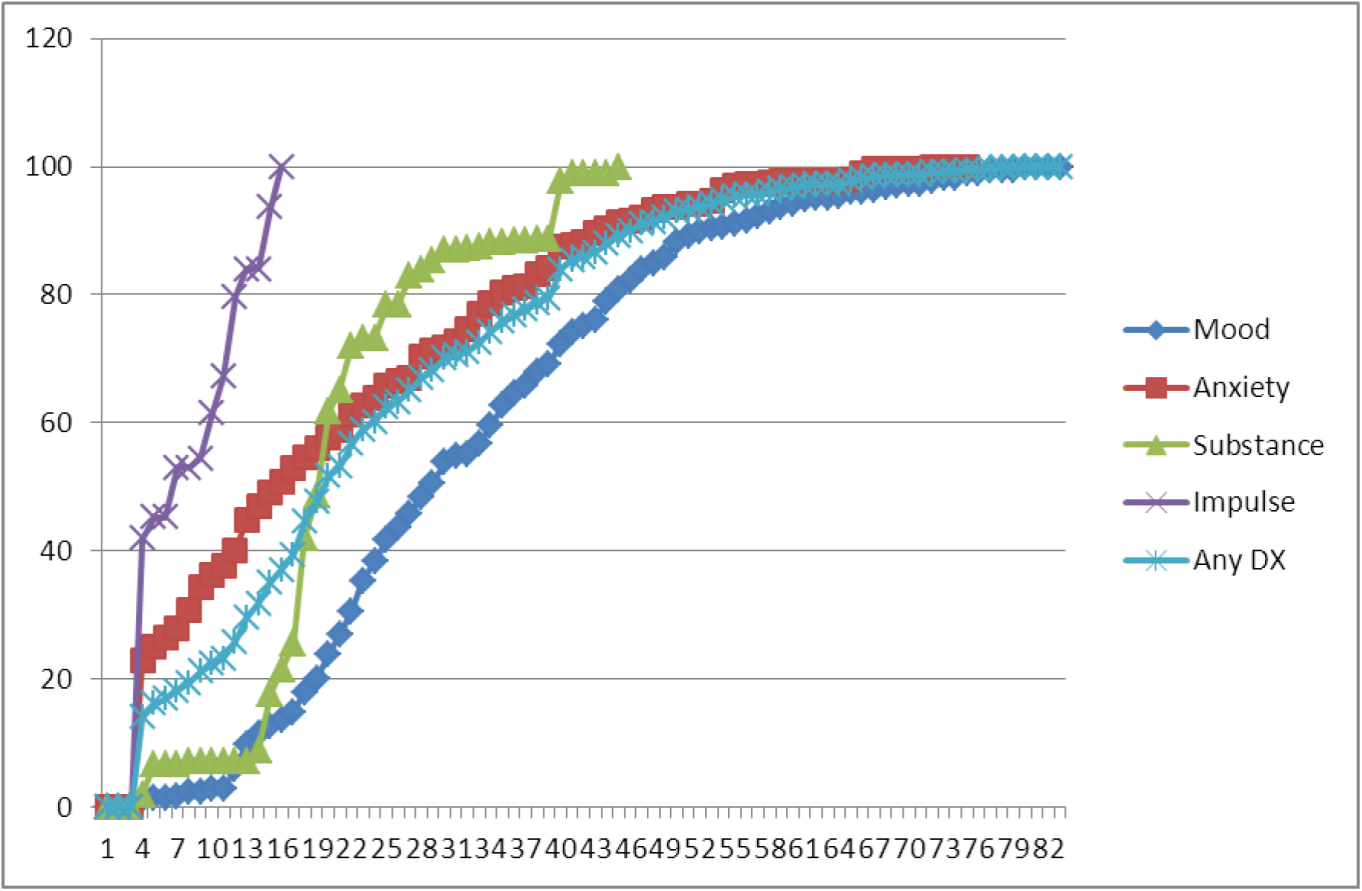
Age-of-onset (AOO) distributions for 4 disorder categories.

Cumulative lifetime risk curves (Fig 2) showed that impulsive disorder had an earlier age of onset than the other three diagnostic categories. The median age of onset was much earlier for any impulsive disorder (age 8 years) compared with substance abuse (21 years), anxiety (26 years) and mood disorders (40 years). Age of onset was concentrated in a very narrow age range for impulsive and substance disorders with interquartile ranges (IQRs) of only 8 (IQRs: 5–13 years) and 9 years (IQRs: 19–28 years), respectively, compared with 27 years (IQRs: 25–52 years) for mood disorders and with 34 years (IQRs: 10–44 years) for anxiety disorders. Disorder specific age-of-onset distribution within diagnostic main categories was similar for impulsive and substance disorders. The age-of-onset distribution of anxiety disorders was more diverse with specific and social phobia having an earlier median age of onset (9 years; IQRs: 5–22 years and 13 years; 5–18 years, respectively) and post-traumatic stress disorder (PTSD) and Generalized Anxiety Disorder (GAD) with later median ages of onset (32 years; IQRs: 20–45 years and 42 years; 30–57 years, respectively). Bipolar disorder had an earlier age of onset (28 years; IQRs: 23–35 years) than major depressive disorder (41years; 26–53 years).

### Prevalence and severity of 12-month mental disorders

The severities of the 12-month prevalence of the four main categories of mental disorders were almost equally distributed (one third) in the three categories of severity (mild, moderate and serious) (Table 4). Significant differences between severity were found in some specific disorders including Social Phobia, Post-Traumatic Stress Disorders and Alcohol abuse without dependence and within the number of disorders. No differences in the severity distribution were found between males and females (Table 4). Substance disorders, Post-traumatic stress and bipolar disorder were the three specific conditions with a higher percentage of respondents with a serious severity.

**Table 4:** Twelve-Month Prevalence and Severity of DSM-IV disorders in PEGASUS-Murcia project (proportions and 95% Confidence Interval).

							Severity+				
				Total			Male			Female	
			Mild	Moderate	Serious	Mild	Moderate	Serious	Mild	Moderate	Serious
DisorderGroup	Disorder	N	% (95% CI)	% (95% CI)	% (95% CI)	% (95% CI)	% (95% CI)	% (95% CI)	% (95% CI)	% (95% CI)	% (95% CI)
**Anxiety Disorder**	Panic disorder*	19	34.4(12.7;65.3)	27.4(13.3;48.2)	38.3(16.5;66.0)	28.4(2.4;86.4)	71.6(13.6;97.6)	0	35.5(12.1;68.6)	19.0(7.6;40.3)	45.5(17.2;77.0)
	Generalized anxiety disorder*	77	23.9(16.3;33.5)	36.7(18.7;59.5)	39.4(19.2;64.0)	14.7(3.3;46.2)	40.9(14.2;74.4)	44.4(10.6;84.3)	26.4(20.2;33.6)	35.6(17.3;59.4)	38.0(18.7;62.1)
	Social phobia*	33	5.8(0.9;28.4)	54.2(36.2;71.2)	40.0(26.8;54.8) †	3.1(0.3;29.0)	68.3(17.2;95.7)	28.6(3.4;81.9)	6.6(0.9;35.4)	50.0(30.0;69.9)	43.5(29.6;58.4)
	Specific phobia*	118	37.5(24.2;52.9)	40.2(25.0;57.5)	22.3(14.1;33.5)	44.6(12.0;82.6)	44.1(10.0;84.8)	11.3(1.2;57.0)	36.1(24.2;50.0)	39.4(26.7;53.9)	24.5(16.5;34.7)
	Agoraphobia without panic*	7	0	23.8(4.4;68.1)	76.2(31.9;95.6)	0	0	0	0	23.8(4.4;68.1)	76.2(31.9;95.6)
	Post-traumatic stress disorder**	26	25.9(10.6;50.8)	9.8(5.7;16.3)	64.3(44.3;80.3) †	51.1(16.9;84.3)	8.0(0.3;71.1)	40.9(7.9;84.8)	13.0(5.2;28.9)	10.7(2.1;40.7)	76.3(64.4;85.2)
	Adult separation anxiety disorder*	0									
	Obsessive Compulsive Disorder***	3	75.5(14.0;98.3)	24.5(1.7;86.0)	0	0	0	0	75.5(14.0;98.3)	24.5(1.7;86.0)	0
	Any anxiety disorder**	227	32.1(29.3;35.0)	37.4(25.2;51.4)	30.6(20.2;43.4)	31.3(21.1;43.7)	44.6(18.9;73.5)	24.1(6.3;60.1)	32.3(27.4;37.5)	35.4(24.1;48.6)	32.4(23.2;43.1)
**Mood Disorder**	Dysthymia*	9	36.8(5.0;86.5)	53.3(11.1;91.3)	9.9(1.4;46.6)	0	84.7(18.6;99.3)	15.3(0.7;81.4)	61.5(12.9;94.5)	32.3(4.3;83.4)	6.3(0.4;50.3)
	Major depressive disorder*	154	32.8(23.4;43.8)	36.9(22.2;54.5)	30.3(18.4;45.6)	19.2(7.2;42.3)	39.6(16.5;68.5)	41.2(17.1;70.4)	37.7(27.7;48.9)	35.9(17.7;59.4)	26.4(13.4;45.4)
	Bipolar Disorder (Broad)*	19	13.2(2.6;46.6)	23.1(10.1;44.5)	63.7(41.6;81.3)	3.1(0.2;29.4)	22.6(4.7;63.4)	74.4(37.9;93.2)	17.5(2.8;60.8)	23.3(6.1;58.6)	59.2(27.2;85.0)
	Any mood disorder*	173	30.7(22.0;41.0)	35.4(21.6;52.2)	33.9(23.0;46.8)	17.3(6.7;37.8)	37.6(16.2;65.3)	45.1(21.6;71.0)	35.6(26.3;46.2)	34.6(17.1;57.6)	29.8(17.6;45.8)
**Impulse Disorder**	Oppositional-defiant disorder**	1	100	0	0	0	0	0	100	0	0
	Conduct disorder**	1	100	0	0	0	0	0	100	0	0
	Attention deficit disorder**	5	61.7(12.3;94.9)	16.5(1.2;76.7)	21.7(2.0;79.4)	64.1(6.8;97.8)	35.9(2.2;93.2)	0	59.7(11.1;94.6)	0	40.3(5.4;88.9)
	Any impulse-control disorder**	6	62.8(13.6;94.8)	16.1(1.2;75.5)	21.1(2.0;78.3)	64.1(6.8;97.8)	35.9(2.2;93.2)	0	61.8(12.7;94.7)	0	38.2(5.3;87.3)
**Substance Disorder**	Alcohol abuse w/ or w/out dependence**	14	39.2(12.5;74.5)	24.8(3.8;73.2)	36.0(10.3;73.5) †	39.3(11.6;76.1)	24.6(3.4;75.0)	36.2(9.9;74.5)	37.9(2.4;93.7)	31.1(7.2;72.3)	31.1(7.2;72.3)
	Alcohol abuse withoutdependence**	12	53.5(19.4;84.5)	33.7(4.9;83.4)	12.8(2.8;42.4)	54.1(18.0;86.4)	33.9(4.4;85.1)	12.0(2.6;41.1)	37.9(2.4;93.7)	31.1(7.2;72.3)	31.1(7.2;72.3)
	Alcohol dependence**	3	0	0	100	0	0	100	0	0	0
	Drug abuse w/ or w/o dependence**	3	0	34.5(9.0;73.7)	65.5(26.3;91.0)	0	21.6(1.1;87.1)	78.4(12.9;98.9)	0	100	0
	Drug abuse without depencence**	1	0	100	0	0	0	0	0	100	0
	Drugdependence**	2	0	21.6(1.1;87.1)	78.4(12.9;98.9)	0	21.6(1.1;87.1)	78.4(12.9;98.9)	0	0	0
	Any substance use disorder**	17	35.2(11.6;69.2)	26.2(5.6;67.9)	38.7(13.1;72.5)	35.8(10.6;72.5)	24.6(4.1;71.5)	39.7(13.1;74.2)	22.3(1.2;87.6)	59.4(20.2;89.5)	18.3(8.5;34.9)
**Any Disorder**	Any**	364	35.2(29.5;41.5)	35.6(24.0;49.1)	29.2(20.8;39.4)	30.0(19.0;44.0)	34.9(17.4;57.7)	35.2(16.6;59.7)	37.3(30.6;44.6)	35.9(22.9;51.4)	26.8(18.2;37.5)
	0 Disorders**	1095	0	0	0	0	0	0	0	0	0
	1 Disorder**	273	42.4(36.3;48.7)	34.6(23.3;47.9)	23.1(14.9;33.8) †	39.9(26.3;55.3)	29.5(15.7;48.5)	30.6(12.9;56.8)	43.4(34.5;52.7)	36.5(23.2;52.2)	20.1(11.2;33.2)
	2 Disorders**	68	15.9(7.7;29.9)	42.3(21.5;66.2)	41.9(21.7;65.1) †	3.3(0.3;25.0)	52.7(13.9;88.5)	44.0(10.3;84.4)	22.7(10.3;42.9)	36.6(15.4;64.6)	40.7(22.5;61.9)
	3+ Disorders**	23	8.5(1.3;40.5)	26.1(8.8;56.3)	65.4(50.0;78.1) †	0	18.8(1.4;78.5)	81.2(21.5;98.6)	9.8(1.5;42.8)	27.2(9.2;57.8)	63.1(48.0;75.9)

Percentages in the three severity columns are repeated as proportions of all cases and sum to 100% across each row. Part 1 Total Sample Size = 2621. Part 2 Total Sample Size = 1459.

+ Severity calculated using part 2 weights. See the “Methods” section text for a description of the coding rules used to define the severity levels.

* Part1 sample, prevalence calculated using part 1 weights.

** Part2 sample, prevalence calculated using part 2 weights.

*** Part3 sample, 33% random draw using part 2 weights.

† p-value ≤ 0.05 for Mild, Moderate and Serious Severity in the Total Sample.

### Socio-demographic predictors

Table 5 shows the multivariate association between the sociodemographic variables and the 12-month prevalence of any disorder, any mood and any anxiety disorders. Women had a significantly higher risk than men for anxiety and mood disorders. Younger people had a higher risk for any disorder than the oldest with a significant linear trend for any disorder and for any anxiety disorder, but not for any mood disorder. There was an increasing risk of those individuals as the level of income becomes lower for any disorder, any mood and any anxiety disorder with a significant linear trend for the first two of them and a tendency for the latest.

**Table 5:** Twelve-month prevalence of any disorder, any mood and anxiety disorders according to socio-demographic variables (adjusted Odds Ratios^&^ and 95% Confidence Interval. (CI)

		Any Disorder			Any Mood Disorder			Any AnxietyDisorder		
Variable	Label	OR ^&^	95% CI	P-value for linear trend	OR^&^	95% CI	P-value for linear trend	OR^&^	95% CI	P-value for linear trend
**Sex**	M	1	.		1	.		1	.	
	F	**3.37**	**2.02; 5.62**		**4.07**	**2.08; 7.93**		**4.06**	**2.62; 6.31**	-
**Age**	18–34	**3.00**	**1.03; 8.71**		3.96	0.53; 29.29		1.87	0.86; 4.06	
	35–49	**2.40**	**1.01; 5.69**		3.48	0.71; 17.07		1.49	0.90; 2.47	
	50–64	2.11	0.95; 4.69		3.09	0.77; 12.45		1.47	0.71; 3.07	
	> 65	1		**0.0127**	1		0.2280	1		**0.0123**
**Family**	Low	**1.93**	**1.34; 2.80**		**2.19**	**1.27; 3.77**		**1.56**	**1.01; 2.40**	
**Income** †	Low-Average	1.83	0.98; 3.41		2.39	0.88; 6.52		1.45	0.87; 2.42	
	High-Average	**1.58**	**1.18; 2.11**		1.70	0.79; 3.66		**1.30**	**1.01; 1.69**	
	High	1		**0.0078**	1		**0.0088**	1		0.1001
**Marital**	Married/Cohabiting	1			1			1		
**Status**	Sep./Widowed/Divorced	1.15	0.47; 2.80		1.23	0.28; 5.39		0.84	0.42; 1.68	
	Never Married	1.34	0.72; 2.50	-	1.88	0.89; 3.96	-	0.97	0.48; 1.97	-
**Education** ^**#**^	None or Primary	1.03	0.55; 1.90		0.92	0.40; 2.14		1.19	0.57; 2.48	
	Basic	0.94	0.51; 1.74		1.07	0.50; 2.28		1.00	0.53; 1.88	
	Secondary	0.73	0.47; 1.15		0.67	0.21; 2.14		0.81	0.53; 1.24	
	College	1		0.7459	1		0.7735	1		0.4532
**Employment**	Working	1			1			1		
	Student	1.40	0.71; 2.75		1.15	0.51; 2.62		1.35	0.49; 3.70	
	Homemaker	1.54	0.99; 2.39		0.92	0.51; 1.65		**1.65**	**1.13; 2.41**	
	Retired/Disabled	1.72	0.86; 3.46		2.06	0.54; 7.82		1.14	0.37; 3.51	
	Unemployed	1.72	0.92; 3.23		1.82	0.66; 5.02		1.02	0.46; 2.27	
	Others	2.53	0.59; 10.79	-	3.05	0.49; 18.92	-	2.61	0.74; 9.16	-
**12-month**	0	1			1			1		
**ECS** ^&^	1	0.71	0.40; 1.26		0.81	0.36; 1.82		0.81	0.43; 1.52	
	2	0.86	0.37; 2.00		1.01	0.40; 2.55		0.94	0.38; 2.29	
	3	**6.88**	**2.44; 19.42**	0.2876	4.83	0.83; 28.28	0.3584	**3.74**	**1.46; 9.56**	0.6514

^**&**^ **Adjusted OR:** all sociodemographic variables are included in the model

† Family income is defined as a four-category income scale calculated as the ratio of family income in the past 12 months divided by the median income for Spain. Low income is defined as less than or equal to 0.5, low average as 0.5 to 1.0, high average as 1.0 to 2.0, and high as over 2.0.

^**#**^ Completed years of education (four categories: None or primary: 0–7 years; Basic: 8–11 years; Secondary: 12–15 years and College: 16 or more years of education)

^&^ 12-month ECS (12-month Economic Crisis Score) as the sum of the score of the three events suffered during the last 12 months. Range value from 0 to 3.

None of the three individual situations (“Have you been unemployed or looking for work for over a month without success?”; “Have you been made redundant?”; and “Have you had a severe financial crises or major economic problems?”) included in the Economic Crisis Score (ECS) had a significant relationship with Any Disorder (OR = 0.63, 95%CI: 0.27 to 1.46; 0.61, 0.23 to 1.63; and 0.74, 0.35 to 1.56, respectively), Any Mood Disorder (1.60, 0.72 to 3.60; 1.37, 0.36 to 5.18; and 1.55, 0.62 to 3.87, respectively) or Any Anxiety Disorder (1.56, 0.74 to 3.30; 1.83, 0.78 to 4.29; and 1.24, 0.67 to 2.31, respectively) (data not shown in Table 5). The cumulative number of economic events showed no significant linear trend. Interestingly, those who had been exposed to the three economic stressful events at the same time during the last 12 months had almost seven times more risk of having any mental disorder during this period of time, specifically related to anxiety disorders. Unadjusted odds ratios of all these variables are presented in S2 Table.

## Discussion

This first analysis of the PEGASUS-Murcia project further emphasize the consequences of the economic crisis in terms of prevalence and severity of mental disorders in a representative sample of the general population of the South-East of Spain. Prevalence was strongly associated with exposure to stressors related to the economic crisis. Economic crisis affects people from all population subgroups, regardless of social standing and occupational status. It also confirms previous data showing that mental disorders are common in the general population, are more frequent in females and moderate-serious cases are almost two thirds in those with a DSM-IV diagnosis during the economic crisis in Murcia. Representative studies of the general population are expensive and complex, so that an economic crisis is not a good period of time to get specific funding to carry them out. The Pegasus-Murcia project offers a great opportunity to analyze related factors that moderate mental disorders in general population.

### Prevalence of mental disorders

These results confirm that mental disorders are common in the general population and approximately one in three respondents reported a lifetime history of any mental disorder according to DSM-IV. The fact that anxiety disorders are more prevalent than mood disorders and the latter are more prevalent than substance disorders is consistent with previous published data [4,24,30,38]. Twelve-month prevalence in Murcia during the period of the economic crisis seem significantly superior of that described ten years before in Spain for Any Disorder (16.3, 95%CI = 13.7 to 19.2 vs 9.2; 7.8 to 10.6 [7], respectively) and for Any Anxiety Disorder (9.7; 7.6 to 12.2 vs 5.9; 4.5 to 7.3[7], respectively). Other specific diagnostic categories present a tendency towards a superior prevalence in our recent survey than in the previous one (Any Mood Disorder, Any Substance Use Disorder, and Any Impulse-Control Disorder). Even though the use of the latest version of the CIDI in the Murcia Survey, which might explain some small part of the differences found, a more plausible explanation, that needs to be confirmed, is related to the impact of the current economic recession. The higher prevalence of mental disorders during a period of crisis has also been described in other studies in different areas of the world affected by an economic recession, such as Hong-Kong [17], Canada [39] and Greece [40]. Indirect measures of mental disorders also point to this impact of the current economic situation in Spain, such as an increase in the prevalence of poor mental health among men [9], an increase in suicidal behavior [10;11] and an increased frequency of mental disorders among primary care attendees [12].

### Age-of-onset, persistence, and severity

The ratio of the 12-month prevalence to lifetime prevalence is considered to be an indirect indicator of persistence or progression of the illness with higher ratios suggesting higher persistence throughout life [29]. Anxiety disorders followed by mood disorders are the more persistent disorders and is consistent with data previously published from Spain and other countries, such as USA and France for example (data calculated from [4,5]). Typically, anxiety disorders begin much earlier than other common mental disorders, including mood and substance use disorders. These age-of-onset distributions are consistent with previous epidemiological surveys [5,41] and confirm that, in general, mental disorders have an early age of onset.

A third of participants with any disorder have a condition with mild, a moderate or a serious severity during the last 12-month mental disorders. This distribution has a higher percentage of people with the higher degree of severity compared to the severity distribution of the Spanish sample were 20.7% were classified as serious, 44.6% as moderate, and 34.8% as mild [42] and the higher proportion with a mild disorder in the international comparison within the WHO-World Mental Health Surveys [4].

### Predictors

Associated factors for mental disorders are consistent with previous community surveys. For females, an inverse association between prevalence and age and higher rates in those with low income are associated with mental disorders. Interestingly, some classical sociodemographic factors, such as being married, level of education and employment were not associated with mental disorders in our sample. Finally, those exposed to three economic adverse events in the 12 months period prior to the interview were at the greatest risk. Stressful Life Events have been shown to be related to mental disorders, specifically to depression [43] and anxiety disorders [44]. Personality traits, such as Neuroticism or Resilience, and different genetic factors might modulate the effect of stressors on mental disorders and these require further analysis.

It is possible that during periods of economic crisis, the effect of some sociodemographic factors associated with mental disorders might be modulated by financial circumstances. For example, economic strain increases familial stress so as being married is not acting as a protective factor against mental disorders [39,40]. Economic stressors related to the current financial recession affecting the general population of a country or a region also have general consequences, independently of educational level and employment status. It is generally accepted that unemployed individuals have poorer mental health than their employed counterparts [45], but temporary employment also induces higher psychological morbidity compared to permanent employment [46]. This situation is increased during periods of economic crisis due to lower income, greater job insecurity, feelings of powerless, lack of promotion, increased workload, and exposure to more difficult work conditions and environmental stress and it may precipitate mental disorders [47]. The negative effects of the recession on mental health may also be extended to employed individuals [48,49], and workers with mental health problems have an increased likelihood for transition into unemployment [50], closing a vicious circle between economic recession, employment status and mental health.

### Limitations

Our results should be interpreted within the context of some limitations. Firstly, the response rate of 67.7% is not entirely satisfactory but is above the 60% conventionally considered as a minimum standard [51] and is above the total participation rate of other studies that participated in the previous ESEMeD project [6], and those interviewed were comparable to those from the available census data of Murcia suggesting our final sample was representative of the general population of the region. It is possible that some patients with mental disorders may have been more reluctant to participate in the survey, thus our results may even be an underestimate the real prevalence of mental disorders in the general population. Secondly, schizophrenia and other non-affective psychoses were not included as a diagnosis because previous validation studies suggested that they tend to be overestimated in lay-administered interviews [4,52,53]. However, these same studies also suggest that the vast majority of respondents with clinician-diagnosed non-affective psychoses meet the criteria for CIDI anxiety, mood, or substance disorders and should be consequently captured as cases even if non-affective psychoses are not assessed. A clinical reappraisal study is currently underway to add new insights to this possibility as those participants at high risk of psychosis are being re-interviewed by a clinical psychiatrist with the module C (Psychotic Disorders) of the SCID [25]. Thirdly, the diagnosis was based on a diagnostic instrument, the CIDI 3.0, with fully structured interviews. The limitation of this instrument to report DSM-IV diagnoses is reduced by evidence of moderate-to-excellent concordance with diagnoses of most mental disorders [54,55]. It has been widely used in previous epidemiologic studies all over the world using similar methodology, therefore enabling national and international comparisons. Fourthly, lifetime diagnoses and age-of-onset estimations were based on retrospective recall and this limitation presumably led to an underestimation of prevalence, over-reporting of persistence, and possible distortion of estimates of correlates, possibly in a differential way across disorders as a function of differences in age at onset [56]. However, the structured design of the diagnostic instrument and the strategy used in the WMH surveys have been shown to diminish this recall bias [57]. Finally, the cross-sectional design while allowing association studies, limits the possible causal interpretation of the findings.

### Clinical implications

Mental disorders are common in the general population and almost two thirds of 12-month DSM-IV cases are considered to be moderate-serious severity, especially during an economic crisis period. This situation has important clinical implications. Firstly, economic crisis affects people from all population subgroups, regardless of social standing and occupational status [14]. Second, during recessions, physicians should be especially mindful of the psychological impact of patients’ economic situation and work environment, including its stability, when diagnosing and treating mental health concerns [49]. Third, intervention programs for unemployed people and for those affected by financial problems should be considered as they may ameliorate economic-related distress among people [58] and reduce the strong association between Mental Disorders and perceived stigma [59]. Finally, economic crisis also might be a window of opportunity to reform and strengthen mental health services by different long term policies rather than nonspecific short term initiatives focused mainly on reducing health costs [14,48,60,61]. Periods of crisis also offer a possibility for better collaboration between professionals from different areas (clinicians, public health researchers, social leaders and policymakers) to develop more efficient approaches to prevent and alleviate the negative impacts of the financial crisis on mental health. Population studies during economic crisis are especially important to contribute to the knowledge of the risk factors involved in mental disorders.

## Supporting Information

S1 Table Specific STROBE Checklist. (DOCX)

S2 Table Twelve-month prevalence of any disorder, any mood and anxiety disorders according to socio-demographic variables (unadjusted Odds Ratios and 95% Confidence Interval. CI). (DOC)
