# Supplementary material for: Prevalence of Mental Disorders in the South-East of Spain, One of the European Regions Most Affected by the Economic Crisis: The Cross-Sectional PEGASUS-Murcia Project
Source: PLoS One. 2015 Sep 22;10(9):e0137293. doi: 10.1371/journal.pone.0137293 (PMC4578930; doi:10.1371/journal.pone.0137293)
Supplement: S2 Table — (DOC) [file pone.0137293.s002.doc]

**S2 Table. Twelve-month prevalence of any disorder, any mood and anxiety disorders according to socio-demographic variables (unadjusted Odds Ratios and 95% Confidence Interval. CI)**

|  | | **Any Disorder** | | | **Any Mood Disorder** | | | **Any Anxiety Disorder** | | |
| --- | --- | --- | --- | --- | --- | --- | --- | --- | --- | --- |
| **Variable** | **Label** | **OR** | **95% CI** | **P-value for**  **linear trend** | **OR** | **95% CI** | **P-value for**  **linear trend** | **OR** | **95% CI** | **P-value for**  **linear trend** |
| **Sex** | M | 1 | - |  | 1 | . |  | 1 | . |  |
|  | F | **2.97** | **2.05 ; 4.30** |  | **3.00** | **1.89 ; 4.77** |  | **4.18** | **2.47 ; 7.07** |  |
| **Age** | 18-34 | 1.71 | 0.78 ; 3.76 |  | 2.44 | 0.78 ; 7.65 |  | 1.23 | 0.62 ; 2.44 |  |
|  | 35-49 | 1.20 | 0.71 ; 2.03 |  | 1.65 | 0.77 ; 3.54 |  | 0.94 | 0.57 ; 1.55 |  |
|  | 50-64 | 1.30 | 0.77 ; 2.18 |  | 1.74 | 0.71 ; 4.26 |  | 1.18 | 0.72 ; 1.94 |  |
|  | > 65 | 1 |  | 0.0882 | 1 |  | 0.0883 | 1 |  | 0.3642 |
| **Family** | Low | **2.18** | **1.42 ; 3.35** |  | **2.45** | **1.62 ; 3.70** |  | 1.64 | 0.98 ; 2.73 |  |
| **Income** † | Low-Average | **1.77** | **1.03 ; 3.04** |  | **2.36** | **1.08 ; 5.17** |  | 1.35 | 0.91 ; 2.01 |  |
|  | High-Average | **1.49** | **1.02 ; 2.16** |  | **1.70** | **1.04 ; 2.79** |  | 1.23 | 0.81 ; 1.87 |  |
|  | High | 1 |  | **0.0037** | 1 |  | **0.0028** | 1 |  | **0.0479** |
| **Marital** | Married/Cohabiting | 1 |  |  | 1 |  |  | 1 |  |  |
| **Status** | Sep./Widowed/Divorced | 1.62 | 0.92 ; 2.86 |  | 1.88 | 0.89 ; 3.98 |  | 1.24 | 0.61 ; 2.49 |  |
|  | Never Married | 1.53 | 0.91 ; 2.57 |  | **2.14** | **1.08 ; 4.25** |  | 1.00 | 0.62 ; 1.62 |  |
| **Education** | None or Primary | 0.98 | 0.50 ; 1.92 |  | 0.76 | 0.28 ; 2.06 |  | 1.20 | 0.60 ; 2.42 |  |
|  | Basic | 0.95 | 0.50 ; 1.80 |  | 0.98 | 0.45 ; 2.12 |  | 1.01 | 0.47 ; 2.15 |  |
|  | Secondary | 0.75 | 0.50 ; 1.14 |  | 0.65 | 0.22 ; 1.92 |  | 0.82 | 0.52 ; 1.30 |  |
|  | College | 1 |  | 0.8086 | 1 |  | 0.8357 | 1 |  | 0.4474 |
| **Employment** | Working | 1 |  |  | 1 |  |  | 1 |  |  |
|  | Student | 2.03 | 0.87 ; 4.73 |  | 1.87 | 0.69 ; 5.13 |  | 1.64 | 0.63 ; 4.27 |  |
|  | Homemaker | **1.95** | **1.18 ; 3.22** |  | 1.11 | 0.69 ; 1.77 |  | **2.70** | **1.61 ; 4.53** |  |
|  | Retired/Disabled | 0.96 | 0.62 ; 1.47 |  | 0.85 | 0.37 ; 1.96 |  | 0.89 | 0.42 ; 1.90 |  |
|  | Unemployed | **2.04** | **1.20 ; 3.47** |  | **2.23** | **1.05 ; 4.75** |  | 1.13 | 0.58 ; 2.21 |  |
|  | Others | 2.40 | 0.66 ; 8.71 |  | 3.17 | 0.67 ; 14.93 |  | 2.53 | 0.66 ; 9.74 |  |
| **ECS**^&^ | 0 | 1 |  |  | 1 |  |  | 1 |  |  |
|  | 1 | 1.00 | 0.67 ; 1.50 |  | 1.20 | 0.70 ; 2.07 |  | 0.95 | 0.63 ; 1.42 |  |
|  | 2 | 0.97 | 0.39 ; 2.41 |  | 1.22 | 0.41 ; 3.69 |  | 0.72 | 0.27 ; 1.87 |  |
|  | 3 | **6.70** | **2.72 ; 16.47** | **0.0371** | 5.06 | 1.46 ; 17.62 | 0.0868 | 2.15 | 0.83 ; 5.61 | 0.9915 |

† Family income is defined as a four-category income scale calculated as the ratio of family income in the past 12 months divided by the median income for Spain. Low income is defined as less than or equal to 0.5, low average as 0.5 to 1.0, high average as 1.0 to 2.0, and high as over 2.0. ^&^  12-month ECS (12-month Economic Crisis Score) as the sum of the score of the three events suffered during the last 12 months. Range value from 0 to 3.
